# The Efficiency of a Uterine Isthmus Tourniquet in Minimizing Blood Loss during a Myomectomy—A Prospective Study

**DOI:** 10.3390/medicina59111979

**Published:** 2023-11-10

**Authors:** Ligia Balulescu, Simona Brasoveanu, Marilena Pirtea, Oana Balint, Aurora Ilian, Dorin Grigoras, Flavius Olaru, Madalin-Marius Margan, Alexandru Alexandru, Laurentiu Pirtea

**Affiliations:** 1 Department of Obstetrics and Gynecology, Victor Babes University of Medicine and Pharmacy, 300041 Timisoara, Romania; ligia.balulescu@umft.ro (L.B.); marilenapirtea@yahoo.com (M.P.); balint.oana@umft.ro (O.B.); aurora1985@yahoo.com (A.I.); grigorasdorin@ymail.com (D.G.); olaru.flavius@umft.ro (F.O.); pirtea.laurentiu@umft.ro (L.P.); 2 Department of Functional Sciences, Discipline of Public Health, Victor Babes University of Medicine and Pharmacy, 300041 Timisoara, Romania; margan.madalin@umft.ro; 3 General Medicine, Victor Babes University of Medicine and Pharmacy, 300041 Timisoara, Romania; alexandru.alexandru@student.umft.ro

**Keywords:** laparoscopic myomectomy, tourniquet, blood loss

## Abstract

*Background and Objectives*: The objective of this study was to assess the effectiveness of using a peri-cervical tourniquet in reducing blood loss during a laparoscopic myomectomy. *Materials and Methods*: This prospective study evaluated the impact of performing a concomitant tourniquet placement during a laparoscopic myomectomy (LM). A total of 60 patients were randomly allocated to one of two groups: 30 patients who underwent an LM with a tourniquet placement (the TLM group) and 30 patients who benefited from a standard LM (the SLM group). This study’s main objective was to evaluate the impact of tourniquet use on perioperative blood loss, which is quantified as the difference in the pre- and postoperative hemoglobin levels (Delta Hb) and the postoperative blood transfusion rate. *Results*: The mean Delta Hb was statistically lower in the TLM group compared to the SLM group: 1.38 g/dL vs. 2.41 g/dL (*p* < 0.001). The rate of postoperative iron perfusion in the TLM group was significantly lower compared to the SLM group (4 vs. 13 patients; *p* = 0.02). All four patients that required a blood transfusion were from the SLM group. On average, the peri-cervical tourniquet fastening time was 10.62 min (between 7 and 15 min), with no significant impact on the overall operative time: 98.50 min for the TLM group compared to 94.66 min for the SLM group. *Conclusions*: Fastening a tourniquet during a laparoscopic myomectomy is a valuable technique to effectively control intraoperative bleeding and enhance surgical outcomes.

## 1. Introduction

The terms fibroid, myoma, and leiomyoma are interchangeable and refer to the most common gynecological tumors. They have high prevalence rates, affecting 70% to 80% of women by the age of 50 [1].

Uterine leiomyomas, also referred to as fibroids or myomas, represent the prevailing benign pelvic tumors among women. They typically remain asymptomatic and are frequently encountered incidentally. However, these tumors possess the potential to generate complications by exerting pressure on or repositioning adjacent pelvic organs. As a consequence, affected individuals may experience a range of symptoms, including pelvic pressure, pain, heightened urinary frequency, constipation, irregular or heavy menstrual bleeding, recurrent miscarriages, infertility, or maternal–fetal outcomes, such as the need for an emergency cesarean section due to a higher percentage of suspected uterine rupture [1,2,3].

A retrospective cohort study showed that an open myomectomy was associated with a higher rate of cesarean deliveries than a laparoscopic myomectomy, with no severe adverse maternal or neonatal outcomes [4]. In our study, all patients were treated through a laparoscopic myomectomy (LM), a minimally invasive procedure that prioritizes both fertility preservation and patient benefits. This technique offers numerous advantages over a traditional laparotomy, including diminished postoperative pain, reduced durations of hospitalization and recovery, and a decreased likelihood of developing adhesions. Consequently, it has become the preferred approach for conducting myomectomies, as affirmed by several studies [5].

Regardless of the surgical method employed, surgeries involving the uterus and myomas can result in significant blood loss due to the abundant blood supply in these tissues. To mitigate the risk of intraoperative bleeding, various techniques have been described in the literature over time. These techniques can be categorized into two main approaches: non-surgical and surgical interventions.

Non-surgical approaches involve interventions such as intramyometrial injections with vasopressin, as well as the utilization of medications like misoprostol or tranexamic acid. These methods aim to minimize bleeding without the need for a surgical intervention [6].

On the other hand, surgical techniques encompass a range of procedures. These include employing a peri-cervical tourniquet, accomplishing a permanent uterine artery occlusion via bipolar coagulation, opting for uterine artery embolization, and employing a relatively recent combined approach involving a laparoscopic myomectomy (LM) coupled with temporary uterine artery occlusion (TUAO) utilizing sutures or clips. These surgical methods have been devised and refined to specifically address the concern of intraoperative bleeding during uterine and myoma surgeries [6].

The main objective of this study was to determine if the surgical technique has a significant impact on the amount of blood loss that occurs during the procedure. This was quantified as the difference between the pre- and postoperative levels of hemoglobin (defined as Delta Hb) through an analysis of the patients’ blood laboratory test results.

The secondary outcomes measured in the study included the following variables: iron perfusion administration, blood transfusions, length of hospitalization, total duration of the operation, and postoperative anemia.

## 2. Materials and Methods

### 2.1. Study Design

This research article was designed as a single-center, prospective randomized study, following the Consolidated Standards of Reporting Trials (CONSORT) guidelines [7,8].

To evaluate the impact of performing a concomitant tourniquet placement during a laparoscopic myomectomy (LM), 60 patients were randomly allocated to one of two groups: 30 patients who underwent an LM with a tourniquet placement (TLM) and 30 patients who benefited from a standard LM (SLM). We measured the hemoglobin levels preoperatively and 24 h after surgery to evaluate the efficiency of a uterine isthmus tourniquet in minimizing blood loss. Randomization was performed via an alternative selection of procedure, regardless of patient demographic characteristics, respectively, with one patient for the TLM group and one patient for the SLM group.

This study was conducted after receiving permission from the Human Ethical Committee of the University of Medicine and Pharmacy “Victor Babes”, Timişoara, Romania (Nr. 74 /28.06.2021) in accordance with ethical standards. It was conducted between the 1 January 2018 and the 31 December 2020 at the Municipal Emergency Clinical Hospital, Timisoara, Romania. Informed consent was obtained by the author from each patient in this study.

### 2.2. Patient Selection

Women with symptomatic uterine leiomyoma who were considered suitable for LM were recruited from the Department of Obstetrics and Gynecology of Municipal Emergency Clinical Hospital Timisoara, Romania.

The inclusion criteria included (a) patients of reproductive age, between 26 and 40 years, with symptomatic fibroids; (b) patients with a preference for laparoscopic myomectomy and their desire to preserve fertility; and (c) patients who had intramural uterine leiomyomas greater than 4 cm in diameter, which also deformed the uterine cavity.

The exclusion criteria included (a) patients who did not agree to the enrollment or did not pass the inclusion criteria (such as being over 40 years old, no preference for fertility preservation, and personal option for hysterectomy); (b) patients with other types of myomas (such as submucosal and subserosal location) or intramural myomas smaller than 4 cm that did not have an impact on the uterine cavity; (c) cases with suspected malignancy; and (d) pregnancy.

### 2.3. Tourniquet Procedure

All surgical interventions were carried out by a solitary and highly skilled surgeon possessing over a decade of expertise in laparoscopic procedures. This standardization ensured consistency in the surgical techniques and reduced potential variations in the outcomes. Prior to the surgery, neither GnRH agonists nor any intra-operative hemostatic drugs, such as vasopressin injection, were administered. Preoperatively, a single dose of an antibiotic was administrated for prophylaxis. The Foley catheter was removed 24 h after surgery in both groups.

Before commencing the surgical procedures, general anesthesia was administered simultaneously with orotracheal intubation.

Abdominal entry was accomplished using a direct trocar entry technique. CO_2_ insufflation was employed to establish a pneumoperitoneum, maintaining a relatively low intra-abdominal pressure of up to 12 mmHg. A single 10 mm trocar was positioned along the midline, 8 cm away from the umbilicus, while two 5 mm trocars were placed on each side of the lower abdomen. The patient was then reclined in a Trendelenburg position.

The single tourniquet refers to a specialized technique where a tourniquet is utilized to block the uterine arteries by applying pressure on the uterine isthmus. To achieve this, a small incision is made in the avascular area of the broad ligament, located on both sides of the uterine isthmus, specifically above the uterine vessels.

**Figure 1:**
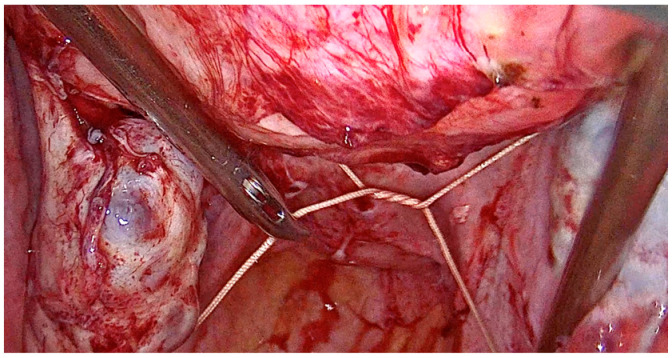
Intraoperative anterior view of laparoscopic tourniquet placement.

**Figure 2:**
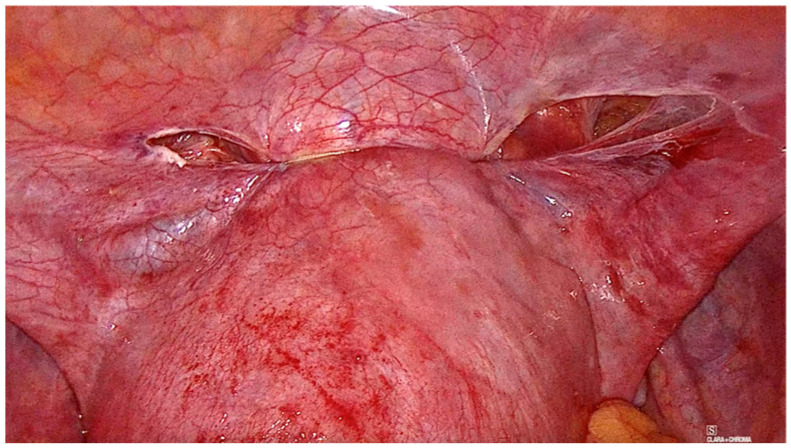
Intraoperative posterior view of laparoscopic tourniquet placement.

The laparoscopic myomectomy with the tourniquet method follows a step-by-step chronological order. First, a homemade tourniquet loop is formed using 1 nonabsorbable thread (Figure 1 and Figure 2). This tourniquet is used to apply pressure and control blood flow to the area during the myoma dissection. Then, the uterine wall is incised, and the myoma/s are located and then removed using traction and countertraction maneuvers. The uterine wall is sutured with a double-layer stitch, utilizing a 29 mm, 3/8 circle needle, and Vicryl 1 thread. Once the uterine wall is closed, the tourniquet loop is carefully removed. The surgical site is thoroughly inspected for any signs of bleeding.

In-bag morcellation is performed in order to remove the fibroid(s) or through a mini-laparotomy using the Alexis system.

### 2.4. Statistical Analysis

To collect and process the data, we utilized the MediFlux 1.1 software environment (developed by ORIGINi^TM^, Amsterdam, The Netherlands). Statistical assessment was performed using Python 3.9.13 programming language (Python Software Foundation). The Pandas library was utilized for data manipulation, and the SciPy library was utilized for statistical analysis.

Descriptive analysis of categorical variables utilized counts and frequency (percentages), while continuous variables were summarized as means.

Shapiro–Wilk test was used to assess the normal distribution of data. When data were normally distributed, the *T*-test was employed to compare means of continuous variables between two groups. For non-normally distributed data, the Mann–Whitney U test was utilized.

For categorical variables, the Chi-squared test was primarily used. In instances where the expected frequencies in the contingency table cells were insufficient, Fisher’s Exact Test was utilized.

Ordinary least squares (OLS) univariate and multivariate linear regression analysis was performed to determine the relationship between the independent variables and the dependent variable. Additionally, statistical tests were conducted to assess the assumptions of the regression models. The Shapiro–Wilk test was used to test the normality of the residuals, the Breusch–Pagan test was employed to evaluate homoscedasticity, and the Durbin–Watson test was utilized to examine the independence of the residuals.

The significance level was set at *p* < 0.05 for all tests.

## 3. Results

The demographic statistics and clinical characteristics of the 60 patients included in the study are presented in Table 1. The patients were divided into two groups in consideration with the performed surgical intervention: 30 (50%) patients in the SLM group and 30 (50%) patients in the TLM group.

There were significant differences observed in age, infertility, parity, and pain. However, the myoma size, number of myomas, bleeding, and preoperative Hb showed no statistically significant differences between the two groups.

The postoperative outcomes (presented in Table 2) showed a significant difference in the hemoglobin loss between the TLM and SLM groups, with a mean loss of 1.38 (1.20–1.57) mg/dL in the TLM group and 2.32 (1.99–2.67) mg/dL in the SLM group (*p* < 0.001). There was also a significant difference in the postoperative Hb, with a mean value of 11.25 (10.78–11.71) mg/dL in the TLM group versus 9.87 (9.30–10.44) mg/dL in the SLM group (*p* < 0.001).

Significant differences were observed in the length of hospitalization. The mean days of hospitalization was significantly shorter in the TLM group, averaging 2.16 (2.02–2.30) days, compared to the SLM group, which averaged 2.64 (2.40–2.88) days (*p* < 0.001).

In terms of postoperative complications, anemia was reported in 5 patients (16.66%) from the TLM group and in 18 patients (60%) from the SLM group (*p* < 0.001).

An iron infusion was performed in 4 patients in the TLM group (13.33%) and in 13 patients in the SLM group (43.33%), showing a significant difference between the two groups (*p* = 0.020). Only in the SLM group, a number of four (13.33%) patients underwent a postoperative blood transfusion. Also, two (6.66%) patients in the SLM group required a laparotomy conversion.

In summary, patients undergoing a TLM surgery experienced limited blood loss and shorter hospitalization days compared to those undergoing SLM. The postoperative complications were similar between the two groups, except for the postoperative blood transfusion, which occurred in four cases in the SLM group, and two cases of laparotomy conversion, which occurred in the same group.

**Figure 3:**
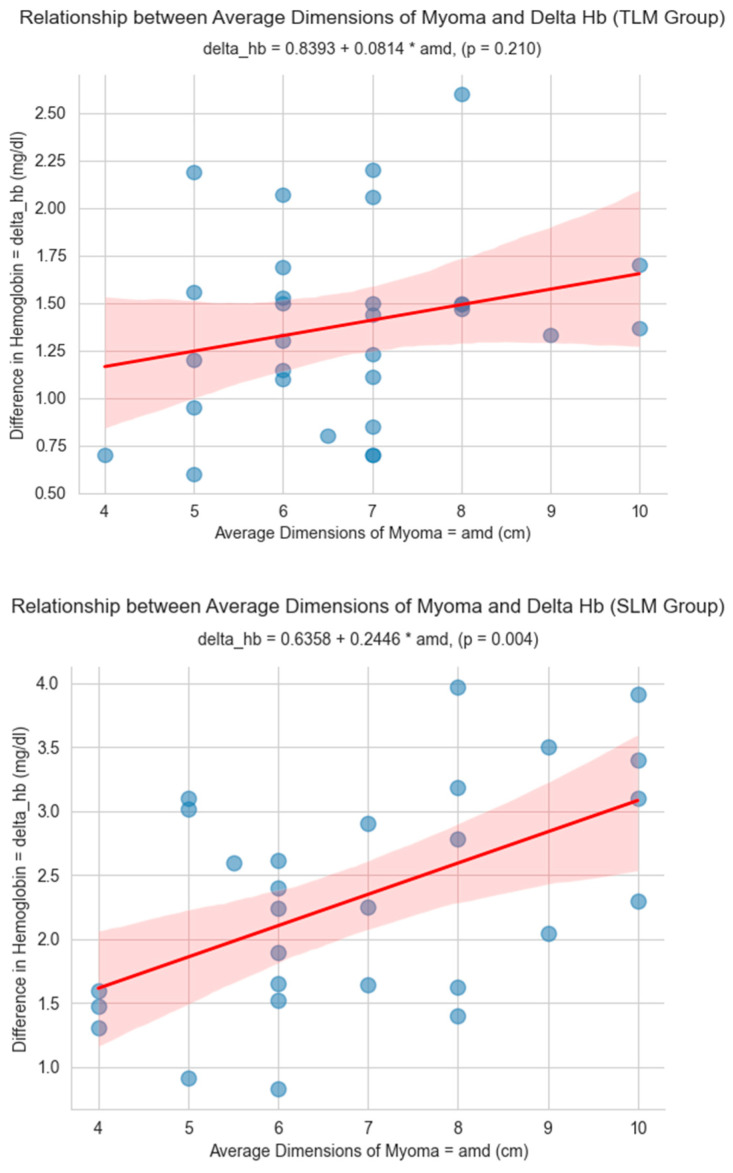
Relationship between average dimensions of myoma and Delta Hb in the two study groups.

A univariate linear regression analysis was performed to investigate the relationship between the differences in the pre- and postoperative hemoglobin levels and the independent variable average dimensions of myoma in two distinct groups: the tourniquet laparoscopic myomectomy (TLM) group and the standard laparoscopic myomectomy (SLM) group (Figure 3).

For the TLM group, the slope value (β = 0.0814) indicated a positive relationship between the Delta Hb and the average dimensions of myoma, but the effect size was relatively small with no statistical significance (*p* = 0.209). In contrast, the SLM group exhibited a stronger positive relationship with a higher slope value (β = 0.2671, *p* < 0.001). In the TLM group, the R-squared value indicated that only about 5.6% of the variation in the Delta Hb could be attributed to changes in the average myoma dimensions, suggesting a weak relationship that was more susceptible to random variability. Conversely, the SLM group had a higher R-squared value of 35.3%, meaning roughly 1/3 of the Delta Hb variation was influenced by myoma dimensions, showcasing a stronger association than in the TLM group.

In conclusion, the comparative analysis of the linear regression models indicated that the relationship between the difference in the hemoglobin levels and the average dimensions of myoma was stronger and more significant in the SLM group than in the TLM group. This preliminarily suggests that the introduction of a tourniquet device during the surgical procedure might counteract the influence exerted by the size of myomas on hemoglobin levels post-surgery.

A multivariate linear regression analysis (presented in Table 3), including age, pain (dyspareunia and/or dysmenorrhea), and bleeding (menorrhagia and/or metrorrhagia) as predictors, supports the same findings. The average dimension of myomas has a statistically significant positive effect (β = 0.240, *p* = 0.009) on the Delta Hb only in the SLM group, which resulted in a higher blood loss in comparison to the TLM group when considering the same dimensions of myomas.

## 4. Discussion

Throughout the past few decades, there have been significant changes in the treatment of fibroids. A laparoscopic myomectomy, a surgical procedure used to remove uterine fibroids through small incisions, was first reported in 1979 [9]. Since then, it has become a popular option for patients who wish to preserve their fertility and retain their uterus for psychological reasons. The advantages of a minimally invasive surgery, such as a quick recovery, low levels of postoperative pain, decreased morbidity, and cosmetic satisfaction, have caused a transition from abdominal myomectomy to LM with the development of laparoscopic surgery. The surgical approach used, however, is determined based on the size and quantity of fibroids as well as the surgeon’s skill.

However, there are some limitations associated with a laparoscopic myomectomy, such as operative blood loss, difficulty in suturing, and locating small myomas through the laparoscope. To address the issue of blood loss during the procedure, various methods have been employed, such as uterotonics, vasopressin, antifibrinolytic drugs, a peri-cervical mechanical tourniquet, laser dissection, temporary cutting of the uterine arteries, misoprostol through the vagina, or the intravenous injection of tranexamic acid or ascorbic acid. Despite these methods, they may have limitations and adverse effects [9].

The article describes a novel technique for a laparoscopic myomectomy (LM) combined with the placement of a tourniquet in the lower segment of the uterus. This innovative approach aims to block blood flow in the ascending branch of the uterine artery, leading to a reduction in intraoperative bleeding during the procedure. The authors emphasize that this technique has the potential to make LM safer and less complicated for patients. The authors underline the lack of studies in the literature on this specific approach of tourniquet.

This study aims to investigate the effectiveness of using the tourniquet in reducing blood loss associated with the surgery. The first outcome measured in our study was operative blood loss as measured by the change in hemoglobin. We found that the surgical use of a tourniquet at the time of an LM compared with an SLM resulted in a statistically significant reduction in the estimated blood loss, expressed as the mean differences in the Hb measured shortly before and on first day after the surgery. The mean differences in Hb (Delta Hb) were statistically significant (1.38(1.20–1.57) vs. 2.32(1.99–2.67), *p* < 0.001)).

Akbaba et al. found that the use of a temporary tourniquet has shown to be statistically effective in reducing hemoglobin loss in patients with multiple large-sized myomas [10], and this suggests that this technique could be a viable option for improving patient outcomes and reducing the need for blood transfusions or other interventions.

In a prospective observational study, the application of the tourniquet was found to significantly diminish blood loss, simplifying the myoma resection process. Notably, 83.3% of patients experienced blood loss below 200 mL, and the rest experienced blood loss between 200 and 400 mL. The use of a tourniquet proves to be a cost-effective, safe, and easily accessible solution, effectively minimizing blood loss during a myomectomy without introducing additional complications to the procedure [11].

The effectiveness of using a tourniquet during surgery in reducing blood loss can be evaluated through various parameters, such as the postoperative drop in hemoglobin, intraoperative blood loss, and the postoperative rate of transfusions. Measuring the difference between preoperative and postoperative hemoglobin levels can provide valuable insight into the amount of blood loss experienced during surgery. While measuring intraoperative blood loss is challenging due to factors like variability in the irrigation fluid, a postoperative Hb drop is a more reliable indicator of the use of a tourniquet in reducing blood loss during the surgery.

A high rate of postoperative blood transfusions can be indicative of significant blood loss during the surgery. The use of a tourniquet that effectively reduces blood loss should be associated with a lower need for transfusions, as the patient’s own blood is better conserved. In our study, the rate of required iron perfusion was 17 (28.33%) in all patients, and the rate of blood transfusions was 4 (6.66%) in all patients. The rate of postoperative iron perfusion in the TLM group was significantly lower compared to the SLM group (4 vs. 13 patients). All four patients that required a blood transfusion were from the SLM group. Other studies also reported a higher incidence of blood transfusions in a standard laparoscopic myomectomy compared to a laparoscopic myomectomy with the use of tourniquet [9,12,13,14].

Uncontrolled bleeding can lead to significant blood loss, potentially resulting in hemodynamic instability and, in severe cases, even a life-threatening hemorrhage. To avoid these complications, sometimes a conversion to laparotomy is needed to ensure proper hemostasis. No patients in the tourniquet group required a conversion to laparotomy, and only two patients in the non-tourniquet group required a conversion to laparotomy.

Regarding the duration of the operation, there was no significant difference between the two groups in our experience: 98.50 (95.89–101.10) min in the TLM group vs. 92.50 (90.11–94.89) min in the SLM group (*p* = 0.095). This is likely due to the fact that the placing of the tourniquet can be performed quickly, between 7 and 15 min, on average in 10.62 min, by an experienced gynecologist.

On the other hand, in the laparoscopic myomectomy group, more time is spent trying to maintain the hemostasis of the sutured wounds of the uterine cavity. Other studies also reported that fastening a tourniquet during a myomectomy is an additional procedure that does not prolong the duration of the surgery [15,16].

The embolization of the uterine artery (UAE), temporary uterine artery clipping, and tourniquet placement are three different techniques used in the context of myomectomy for the treatment of uterine fibroids. Each method has its own advantages and disadvantages, and the choice between them depends on various factors, including the patient’s condition, the size and location of the fibroids, and the surgeon’s expertise. A uterine artery embolization does not involve a major surgery, which means shorter hospital stays and faster recovery times compared to a traditional myomectomy. Since it is a non-surgical approach, blood loss during the procedure is minimal. However, a UAE may not be as effective for large fibroids or those located deep within the uterine wall. While a UAE is generally safe, there are risks of infection, allergic reactions to the embolic agents, and potential damage to nearby organs. The fibroid recurrence rate after the embolization was 17.2% [17].

Artery uterine clipping allows for the surgeon to control the blood flow to the fibroids, reducing bleeding during the myomectomy. Dubuisson et al. conducted a literature review to define the role of preventive uterine artery occlusion during a laparoscopic myomectomy. They reported that six of eight comparative studies showed a substantial decrease in operative blood loss in patients who underwent preventive a uterine artery occlusion during surgery. Temporary clipping has a lower risk of ischemia, minimizing the risk of tissue damage. However, these vascular clamping techniques with vascular clips require the surgeon to undergo a learning curve for operative skills, such as opening the retroperitoneum and identifying the pelvic vasculature [18]. Theoretically, there is a risk of causing damage to the uterine artery during the dissection. In his study, Voss did not observe any risk of organ damage with the opening of the pararectal fossa [19].

This article describes an efficient method to minimize blood loss during a conservative uterine surgery. Other types of surgical uterine-sparing procedures such as caesarean scar ectopic pregnancy [20], uterine arteriovenous fistulas, or interstitial cornual pregnancies [21] can also benefit from the use of a tourniquet to minimize intraoperative bleeding. Also, the method using a tourniquet can improve the surgical effect in cases of severe placenta accreta [22] or can control excessive bleeding in cesarean sections [23].

Overall, these findings suggest that the use of a tourniquet during a laparoscopic myomectomy could be a viable option for improving patient outcomes and reducing complications related to blood loss during the procedure. Applying a tourniquet is a straightforward technique that does not require advanced surgical skills or specialized equipment. By minimizing bleeding, the surgeon can have a clearer view of the surgical site. It is important to consider these aspects when interpreting the results of the studies and to be cautious about drawing strong conclusions based on incomplete or potentially biased data.

Our investigation has some limitations, including the facts that it used a single-center design and all procedures were performed by a single team. Additionally, the hospital’s protocol limited the blood loss estimation to measuring differences in the hemoglobin levels. However, our study’s strengths lie in its prospective design. Notably, we maintained consistency in the surgical approach, except for the tourniquet technique, which we introduced to limit blood loss. This novel technique holds great promise and offers numerous potential applications for uterine-sparing procedures, making it a key strength of our study.

## 5. Conclusions

Fastening a tourniquet during a laparoscopic myomectomy is a valuable technique to effectively control intraoperative bleeding and enhance surgical outcomes. Uterine fibroids, especially multiple or large ones, present unique challenges during surgery, and controlling operative bleeding is critical to avoid complications and ensure patient safety.

Preventive tourniquet use during a myomectomy can have several benefits, including minimizing bleeding during the procedure, lowering the rate of postoperative iron perfusion, decreasing the need for blood transfusions, and shortening the length of the hospital stay. Further research with larger sample sizes would help to validate these results and explore the long-term benefits of this technique.

## Figures and Tables

**Table 1:** Clinical characteristics of the study participants.

Parameter	Mean (Min-Max) or N (%)			
	Study Group N = 60	TLM Group N_1_ = 30 (50%)	SLM Group N_2_ = 30 (50%)	*p*-Value
Age	33.16 (32.10–34.12)	32.36 (30.82–33.90)	33.86 (32.52–35.21)	0.009 ^1^
Infertility	26 (43.33%)	18 (60%)	8 (26.67%) *	0.037 ^2^
Parity	33 (55%)	12 (40%)	21 (70%) *	0.019 ^2^
Myoma size	6.91 (6.46–7.36)	6.71 (6.18–6.25)	7.11 (6.36–7.86)	0.519 ^1^
Number of myomas	1.08 (1–2)	1.1 (1–2)	1.06 (1–2)	0.654 ^1^
Pain	30 (50%)	10 (33.33%)	20 (66.66%)	0.009 ^2^
Bleeding	35 (58.33%)	14 (46.66%)	21 (70%)	0.667 ^2^
Preoperative Hb	12.34 (12.01–12.68)	12.64 (12.18–13.09)	12.05 (11.56–12.55)	0.080 ^3^

^1^ Mann–Whitney U test, ^2^ Chi-squared test, ^3^ *T*-test. * For one case, infertility and parity were not reported.

**Table 2:** Postoperative outcomes of study participants.

Parameter	Mean (SD) or N (%)			
	Study Group N = 58	TLM Group N_1_ = 30 (51.72%)	SLM Group * N_2_ = 28 (48.28%)	*p*-Value
Postoperative Hb	10.58 (10.18–10.98)	11.25 (10.78–11.71)	9.87 (9.30–10.44)	<0.001 ^1^
Delta Hb	1.84 (1.62–2.06)	1.38 (1.20–1.57)	2.32 (1.99–2.67)	<0.001 ^2^
Surgery length	95.60 (93.71–97.50)	98.50 (95.89–101.10)	92.50 (90.11–94.89)	0.009 ^2^
Length of hospitalization	2.40 (2.25–2.54)	2.16 (2.02–2.30)	2.64 (2.40–2.88)	<0.001 ^2^
Anemia postoperative	23 (38.33%)	5 (16.66%)	18 (60%)	<0.001 ^3^
Iron perfusion	17 (28.33%)	4 (13.33%)	13 (43.33%)	0.020 ^4^
Postoperative Blood Transfusion	4 (6.66%)	0 (0%)	4 (13.33%)	-

^1^ *T*-test, ^2^ Mann–Whitney U Test, ^3^ Chi-squared test, ^4^ Fisher’s Exact Test. * The 2 cases with conversion to classical laparotomy were excluded.

**Table 3:** Correlation between selected risk factors and Delta Hb in the two study groups using multivariate linear regression.

Variables	TLM Group		SLM Group	
	Regression Coefficient (β)	*p*-Value	Regression Coefficient (β)	*p*-Value
Age	−0.034	0.119	0.002	0.955
Average Dimension of Myomas	0.068	0.269	0.240	0.009
Pain (Dyspareunia/Dysmenorrhea)	−0.328	0.125	0.070	0.838
Bleeding (Meno/Metrorrhagia)	0.346	0.081	0.035	0.918

## Data Availability

Data are available from the correspondence author, upon reasonable request.
